# Editorial: What We See Through Cardiac Imaging

**DOI:** 10.3390/jcm14196824

**Published:** 2025-09-26

**Authors:** Valeria Pergola, Martina Perazzolo Marra

**Affiliations:** Cardiology Unit, Cardio-Thoracic-Vascular, and Public Health Department, Padova University Hospital, 35128 Padova, Italy; martina.perazzolomarra@unipd.it

The evolving landscape of cardiac imaging continues to redefine our ability to diagnose, stratify risk, and monitor cardiomyopathies. In both congenital and acquired conditions, structural, functional, and dynamic myocardial insights are no longer ancillary; they are foundational. This Special Issue, “What We See through Cardiac Imaging”, assembles diverse contributions reflecting the growing role of multimodality imaging (MMI) as both a diagnostic and prognostic cornerstone.

In patients with systemic right ventricle (sRV) physiology, such as those with transposition of the great arteries (TGA), post-atrial switch, or congenitally corrected TGA (ccTGA), subpulmonary left ventricular (LV) function has historically received limited attention. Piana et al. [1] demonstrate that LV global longitudinal strain (GLS), measured by cardiac magnetic resonance feature tracking (CMR-FT), predicts adverse outcomes in this group, confirming the prognostic significance of this neglected chamber. While the intra-vendor reproducibility of the strain is good [2], inter-vendor variability remains a significant barrier to widespread adoption. Standardization of acquisition, post-processing, and threshold values is essential to ensure reliable surveillance. A related challenge is addressed in patients with repaired aortic coarctation [3]. This supports earlier observations [4] that anatomical severity alone does not predict functional limitation. Integration of exercise testing with cross-sectional imaging and longitudinal outcomes will help define intervention thresholds with greater precision.

Coronary artery anatomy shows considerable variability, ranging from benign variants to anomalies causing hemodynamic compromise or sudden cardiac death (SCD). Baz [5] provides a structured review of coronary artery anomalies (CAAs) and emphasizes CT coronary angiography (CTCA) as the reference non-invasive modality for detailed anatomical assessment. Recent advances in cardiac CT technology have enabled high-quality imaging without ECG synchronization, improving efficiency and reducing patient burden. These early results suggest a promising diagnostic tool, though its applicability may be limited in patients with arrhythmias or very high heart rates, warranting further investigation [6]. Hardware innovations in computed tomography are already enabling shorter and simpler diagnostic workflows. On the other hand, the convergence of artificial intelligence and the metaverse opens scenarios in which the patient becomes an active participant in the diagnostic experience. The near-perfect accuracy achieved by the ICA-based algorithm, further discussed in immersive VR environments, suggests that the future is not only about streamlining procedures but also about reshaping the doctor–patient relationship in a participatory and interactive way [7].

Galzerano et al. [8] highlight the integration of three-dimensional echocardiography, myocardial strain, and vortex dynamics as a promising approach for heart failure assessment, with the potential to uncover subtle ventricular dysfunction before clinical decline. In this context, vortex analysis has also been applied to the athlete’s heart, where it complements morphological remodeling by demonstrating higher energetic parameters in highly trained individuals, thereby reflecting the effects of training intensity and energy consumption [9]. While promising, its prognostic and therapeutic impact remains to be established in longitudinal studies.

Postoperative paradoxical septum (POPS) is a frequent but usually benign finding after cardiac surgery, characterized by preserved LV function, normal perfusion, and absence of injury. Di Virgilio et al. [10] review fifty years of hypotheses, culminating in a heuristic model in which POPS arises from the geometric realignment of ventricular anchor points rather than myocardial damage. This reframes POPS as a functional adaptation rather than pathology. Historical work by Weyman et al. [11] links paradoxical septal motion to RV volume overload, but Di Virgilio et al. [10] integrate past and present evidence into a unified geometric-shift model. While compelling, it remains unproven; prospective, quantitative imaging studies—particularly using 3D deformation analysis—are needed to validate its diagnostic and prognostic relevance.

An extensive review of the Ross procedure by Galzerano et al. [12] underscores its growing recognition, despite a current class IIb guideline status, for offering superior survival, fewer valve-related complications, and better quality of life compared with conventional prostheses in young patients. Consistently, analysis of over 2000 adolescents and young adults from the ECCDB demonstrated excellent outcomes with the Ross procedure, showing the lowest mortality rate (0.4%) and reinforcing its value as a durable and safe option in this challenging population [13]. While the Ross procedure appears to be a compelling option for carefully selected patients, questions remain over long-term (>20 year) autograft durability and optimal homograft preservation. Coordinated, prospective multicenter studies with standardized imaging follow-up are essential to define candidacy and refine guidelines.

Pergola et al. [14] argue for moving beyond a simple ejection-fraction–based classification of heart failure, advocating instead for an integrated multimodality approach that reflects the dynamic and patient-specific nature of the condition. In line with this perspective, a recent comprehensive review of early-stage HFpEF emphasizes the challenges of timely diagnosis, the importance of advanced imaging, and the need for early intervention to reduce progression to overt and advanced disease [15]. MMI is pivotal for candidate selection, surgical planning, and complication detection, including RV failure, thrombosis, valvular dysfunction, and device malposition. While MMI clearly enhances peri- and post-operative assessment, prospective multicenter trials and standardized imaging protocols are needed to confirm survival and quality-of-life benefits [16].

Moving to CTCA, Napoli et al. [17] highlight epicardial and pericoronary adipose tissue (EAT/PCAT) as active contributors to coronary inflammation, plaque vulnerability, and acute coronary syndromes. They position PCAT-CT attenuation as a non-invasive biomarker of coronary inflammation with prognostic potential in CAD. Previous studies [18] showed that in angiographically non-obstructive coronary syndromes, PCAT demonstrates a transient but measurable inflammatory phenotype on CCTA via the pericoronary fat attenuation index (pFAI). Consistently, the large ORFAN study demonstrated that the perivascular fat attenuation index (FAI) robustly predicts cardiac mortality and MACE independently of traditional risk factors and CAD burden, with AI-enhanced risk stratification further refining prognostic accuracy, particularly in patients without obstructive disease [19]. These findings support a paradigm shift toward inflammation-based risk assessment. Still, validation in larger, diverse cohorts and trials linking PCAT modulation to outcome improvements are needed before clinical integration.

Finally, Sonaglioni et al. [20] report a rare, isolated RV thrombosis post-cardiac surgery, initially misdiagnosed as tumor. The case illustrates the diagnostic pitfalls of intracardiac masses and the value of multimodality imaging—and particularly echocardiography—in guiding timely intervention. Extending this diagnostic framework, pulsed-wave tissue Doppler imaging has been proposed for distinguishing pathological right atrial masses from benign pseudomasses such as a prominent crista terminalis or the Chiari network [21]. While promising for embolic risk stratification, its clinical adoption is limited by scarce validation. Multicenter studies are needed to determine its sensitivity, specificity, and reproducibility.

Taken together, the contributions in this Special Issue reinforce the central message: Cardiac imaging has moved beyond anatomy to become a predictive, therapeutic-shaping tool. Across congenital, structural, coronary, and heart failure domains, MMI enables earlier disease detection, sharper risk stratification, and tailored interventions. The challenge ahead is to translate these promising approaches into standardized, evidence-based protocols, validated by robust multicenter trials, to ensure that they benefit the broad patient populations who stand to gain from them.
